# Alcohol consumption is associated with reduced creatine levels in the hippocampus of older adults

**DOI:** 10.1016/j.pscychresns.2019.111019

**Published:** 2020-01-30

**Authors:** Naiara Demnitz, Anya Topiwala, Enikő Zsoldos, Charlotte J. Stagg, Uzay E. Emir, Heidi Johansen-Berg, Klaus P. Ebmeier, Claire E Sexton

**Affiliations:** a Department of Psychiatry, University of Oxford, Oxford, UK; b Wellcome Centre for Integrative Neuroimaging, FMRIB, Nuffield Department of Clinical Neurosciences, University of Oxford, Oxford, UK; c School of Health Sciences, Purdue University, West Lafayette, IN, USA; d Global Brain Health Institute, Department of Neurology, University of California San Francisco, San Francisco, California, USA

**Keywords:** Ageing, MRS, Metabolites, Drinking, Choline

## Abstract

Besides its well established susceptibility to ageing, the hippocampus has also been shown to be affected by alcohol consumption. Proton spectroscopy (^1^H-MRS) of the hippocampus, particularly at high-field 7T MRI, may further our understanding of these associations. Here, we aimed to examine how hippocampal metabolites varied with age and alcohol consumption. Hippocampal metabolite spectra were acquired in 37 older adults using 7T ^1^H-MRS, from which we determined the absolute concentration of N-acetylaspartate (NAA), creatine, choline, myo-inositol, glutamate and glutamine. Thirty participants (mean age = 70.4 ± 4.7 years) also had self-reported data on weekly alcohol consumption. Total choline inversely correlated with age, although this did not survive multiple comparisons correction. Crucially, adults with a higher weekly alcohol consumption had significantly lower levels of creatine, suggesting a deficit in their hippocampal metabolism. These findings add to an increasing body of evidence linking alcohol to hippocampal function.

## Highlights

• Alcohol consumption has previously been associated with hippocampal volume.
• ^1^H-MRS was used to quantify hippocampal metabolite concentrations in older adults.
• Alcohol consumption was negetively correlated with hippocampal creatine levels.
• Future MRS studies may wish to account for drinking variables in their analyses.
• Creatine may not be an apt denominator for metabolite ratios in age-related studies.

## 1 Introduction

Ageing is associated with accelerated atrophy of the hippocampus, with rates affected by a range of modifiable and non-modifiable factors (Fotuhi et al., 2012). One such factor is alcohol, which has been shown to have detrimental effects on the hippocampus (Wilson et al., 2017), with even moderate alcohol consumption associated with hippocampal atrophy (Topiwala et al., 2017).

However, although structural MR can precisely quantify atrophy, it is insensitive to the abnormalities in neuronal metabolism that precede cell death. Proton magnetic resonance spectroscopy (^1^H-MRS) enables the non-invasive measurement of regional metabolite concentrations. The most widely studied metabolites include N-acetylaspartate (NAA), creatine (Cr), and choline (Cho). While NAA and Cho are argued to, respectively, serve as indirect markers of neuronal viability and membrane turnover, creatine plays a pivotal role in cell energy homoeostasis (Rae, 2014). However, despite the sensitivity of MRS to neurodegenerative diseases (Gao and Barker, 2014), and despite the hippocampus occupying centre-stage in the ageing literature, very few studies have applied MRS to examine variations in hippocampal metabolites in older adults.

A recent systematic review of 42 MRS studies examining the relationship between age and metabolites identified just 6 studies of metabolite concentrations in the hippocampus in older adults (aged 65+) (Cleeland et al., 2019). The most consistent finding, observed in 4 studies, was a decrease in NAA or NAA/Cr with age (Angelie et al., 2001; Driscoll et al., 2003; Schuff et al., 1999; Szentkuti et al., 2004), although the opposite relationship has also been reported (Chiu et al., 2014).

In relation to alcohol consumption, proton MRS has not been applied to the hippocampus of older adults to date. Elsewhere in the brain, the direction of changes in metabolite concentrations associated with alcohol consumption remains unsettled, and may be dependant on the region of interest. In alcohol dependant patients, reduced levels of choline and NAA have been reported in the frontal lobe, medial temporal lobe, cerebellum and thalamus (Buhler and Mann, 2011). Levels of Cr and Cho have been found to be higher in the anterior cingulate cortex (Lee et al., 2007) and reduced in frontal white matter (Tunc-Skarka et al., 2015), in association with alcohol use. Although observational studies cannot provide causal evidence, studies from animals suggest these metabolite changes may occur as a consequence of alcohol consumption and, interestingly, that some of these changes may be reversible. In rodents, decreased levels of tCr following alcohol exposure have been shown to return to baseline after withdrawing alcohol (Zahr et al., 2010) – a finding which has been replicated in alcohol-dependant patients (Mon et al., 2012). Taken together, the literature suggests that alcohol consumption, even at moderate quantities (Tunc-Skarka et al., 2015), has broad neurochemical consequences. The directionality of these effects within the ageing hippocampus still remains uncertain, however.

The paucity of hippocampal MRS studies is largely due to the difficulty in obtaining high-quality MRS data from this small, susceptibility-prone, brain region (Bednarik et al., 2015). However, the increased signal-to-noise and spectral resolution afforded by ultra-high field machines means that some of these concerns can be overcome using 7T MRI. Here, we conducted a single voxel ^1^H-MRS study, using 7T imaging, in the right hippocampus of 37 older adults, in order to examine (1) the association between hippocampal metabolites and age and (2) whether our previously observed reduction in hippocampal volume with alcohol consumption might be associated with variations in metabolite concentrations. We hypothesised that, in line with the detrimental effects of ageing and alcohol consumption on hippocampal volume, these variables would be negatively correlated with hippocampal metabolite concentrations.

## 2 Methods

### 2.2 Participants

The Whitehall II Study is a prospective cohort study of British civil servants established in 1985. Between 2012 and 2016, the Whitehall II Imaging Sub-Study randomly selected 800 participants from the Whitehall II Study for an additional assessment phase, which consisted of a 3T MRI brain scan and health assessments at the Wellcome Centre for Integrative Neuroimaging, University of Oxford (Filippini et al., 2014). Participants scanned between 2014 and 2016, who had tolerated the 3T MRI scan well and had no safety contraindications for a 7T scan, were invited for a second visit to undergo a 7T MRI scan, up to 3 months after the 3T MRI scan. Forty-four consenting participants of the Imaging Sub-Study (henceforth the parent cohort) were included in the 7T MRI Sub-Study*,* 37 of which underwent hippocampal MRS. Ethical approval for the Whitehall II Imaging Sub-Study, and the subsequent Whitehall II 7T MRI Sub-Study, was obtained from the Oxford Central University Research Ethics Committee and informed written consent was obtained from all participants.

### 2.3 Sample characteristics

Age, sex and years of full-time education were recorded in a self-report questionnaire.

Current alcohol consumption (units drunk per week) was calculated based on self-report. As part of the Imaging Sub-Study (<3 months before 7T MRI visit), participants were asked to indicate how frequently they consumed each of the following alcoholic drinks in a typical week: small (125 ml), standard (175 ml) or large (250 ml) glass of wine, pint of lower-strength lager/beer/cider (approx. alcohol by volume, or ABV, 3.6%), pint of higher-strength lager/beer/cider (approx. ABV 5.2%), bottle of lager/beer/cider (approx. ABV 5%), can of lager/beer/cider (approx. ABV 5%), alcopop (ABV 5.5%), single small shot of spirits, other (to be specified). The volume of the drink (in ml) was then multiplied by its percentage ABV and divided by 1000 to obtain the UK units of alcohol in that drink, where 1 unit equals 8 gs of ethanol (Department of Health, 1995). Finally, the units of alcohol in one drink were multiplied by frequency in order to obtain the number of UK alcohol units (henceforth units) consumed per week.

### 2.4 Magnetic resonance imaging

Magnetic resonance imaging (MRI) data were acquired on a 7 Tesla Siemens MRI scanner, using a 32-channel head coil, at the Wellcome Centre for Integrative Neuroimaging (WIN), University of Oxford. T1-weighted structural images were acquired to inform MRS voxel placement and tissue content within the voxel (dimension = 0.6 mm^3^, repetition time = 2200 ms, echo time = 3.04 ms echo time, flip angle = 7˚, field of view = 232 mm). To obtain the tissue composition of the MRS voxel, FMRIB's automated segmentation tool (FAST) was applied (Zhang et al., 2001).

### 2.5 Magnetic resonance spectroscopy

Single-voxel MRS data were acquired from a 3.36 mL (10 × 12 × 28 mm^3^) voxel region of interest (VOI) placed over the right hippocampus. Barium titanate pads were positioned along the temporal lobes in order to increase B1 efficiency in the voxel of interest (Lemke et al., 2015). The spectra were obtained using a semi-adiabatic localization by adiabatic selective refocusing (semi-LASER) sequence (Oz and Tkac, 2011; van de Bank et al., 2015) with VAPOUR (VAriable Power RF pulses with Optimized Relaxation delays; (Tkac et al., 2001)) water suppression (TE=36 s, TR=5950 ms, 112 averages, acquisition time=11 min 12 s). In addition, unsuppressed water spectra were acquired from the same volume of interest and used to remove residual eddy current effects and to reconstruct the phased array spectra. Absolute neurochemical concentrations were extracted from the spectra using water signal as an internal concentration reference. Brain metabolites observed by MRS were quantified in the millimolar concentration range, relative to the unsuppressed water signal.

### 2.6 MRS processing and quantification

All MRS data were processed using the LCModel (Provencher, 2001). In order to correct for the effect of cerebrospinal fluid (CSF) on the estimated metabolite concentrations (Quadrelli et al., 2016), CSF content (%) within each VOI was inputted in the LCModel. Using the unsuppressed water signal as internal reference, the following metabolite concentrations were estimated: N-acetylaspartate (NAA), N-acetylaspartylglutamate (NAAG), glutamate (Glu), glutamine (Gln), creatine (Cr), phosphocreatine (PCr), Glycerophosphocholine (GPC), phosphocholine (PCho), GABA, lactate (Lac), aspartate (Asp) and myo-inositol (mI). Total NAA (tNAA; NAA + NAAG), total choline (tCho; PCho + GPC) and total creatine (tCr; Cr + PCr) were computed from their respective components.

All included spectra had Full Width Half Maximum (FWHM) ≤ 17.5 Hz (0.06 ppm). In line with previous reports, metabolites quantified with Cramer-Rao Lower Bounds > 50% were deemed to be unreliable estimates (Voets et al., 2017). GABA, lactate, alanine, glucose, and aspartate could not be reliably measured in over 40% of the participants and were thus excluded from further analyses.

Metabolite concentrations are typically reported as ratios to creatine, assuming the tCr in the voxel is 8uM/g. However, the interpretability of these ratios is clouded by previous reports of increases (Suri et al., 2017) and decreases (Eylers et al., 2016) of creatine levels with age. Accordingly, absolute metabolite concentrations were used in our analyses. Nonetheless, given the frequent use of ratios in the literature, we also present results as ratios to creatine to facilitate comparability with existing reports (Appendix 1).

### 2.7 Statistical analyses

Means and standard deviations are presented for all sample characteristics and metabolite concentrations. Pearson correlations were computed to test the relationship between metabolite concentrations and age. We then examined the relationship between metabolites and alcohol consumption using partial correlations accounting for the effect of age. To account for the 6 neurochemicals being analysed, the threshold for significance was Bonferroni-corrected to *p* = 0.008. All statistical analyses were performed in R version 3.5.1 with RStudio version 1.1.463 (RStudio Team, 2016) using the psych (Revelle, 2018) and ggplot2 packages (Wickham and Sievert, 2016).

## 3 Results

**Fig. 1:**
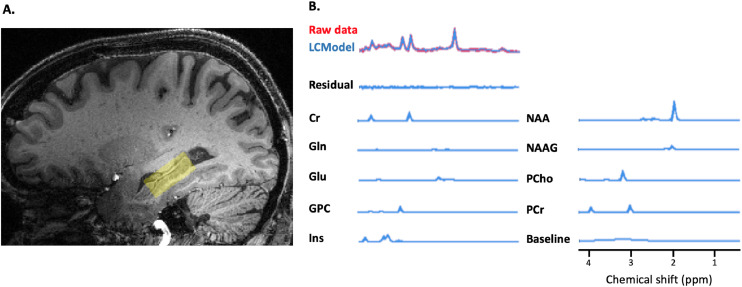
(a) MRS voxel placement on the right hippocampal region overlaid on a T1 image and (b) a representative acquisition from a sample participant, including model fit.

Hippocampal MRS was acquired from thirty-seven participants (Fig. 1). Spectra from six participants were discarded due to lipid contamination and voxel placement. In addition, one participant reported alcohol consumption 3 SDs above the mean and was thus deemed an outlier and excluded from further analyses. This resulted in a final sample of 31 participants (30 for alcohol analyses). Other than having completed more years of full-time education, our sample was, on average, representative of the parent cohort from which it was sampled (Appendix 2). Our sample was mostly male (90.4%), cognitively healthy (mean MoCA = 27.4), and on average had pursued higher education (16 ± 2.9 years of full-time education; Table 1). Age and alcohol were not significantly correlated (*r* = −0.03, *p* = 0.87).

**Table 1:** Overview of sample characteristics.

*n* = 31	Mean ± SD
Age	70.4 ± 4.7
Women (n,%)	3, 9.6%
Education (years)	16 ± 2.9
MoCA	27.4 ± 2.1
Alcohol consumption (units/week)	16.1 ± 15.2

**Fig. 2:**
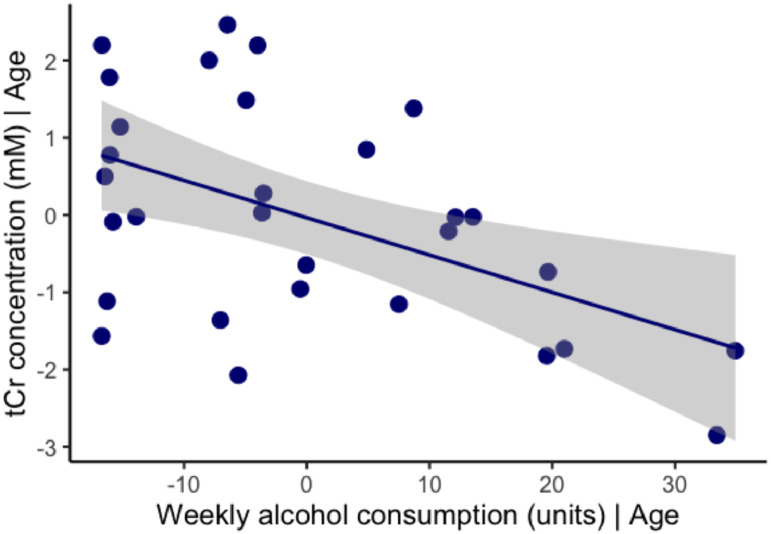
We observed a significant negative correlation between weekly alcohol consumption and [tCr] concentration in the hippocampus, after adjusting for age. Plots of the first order regressions between [tCr] and alcohol consumption and [tCr] and age are available in Appendix 3.

Absolute concentrations of total NAA, total creatine, myo-inositol, total choline, glutamine and glutamate are presented in Table 2. Age was not significantly associated with any metabolite after correction for multiple comparisons. In partial correlations controlling for age, alcohol consumption was negatively correlated with total creatine concentration in the hippocampus (Fig. 2). No other association was observed between metabolites and alcohol consumption (Table 2). Zero-order correlations between metabolite levels and alcohol consumption are presented in Appendix 3.

**Table 2:** Metabolite concentrations and tissue content within the voxel are expressed as mean ± standard deviations. For each metabolite, correlations with age (univariate) and weekly alcohol consumption (bivariate; covariate = age) are shown. Significance level was set at *p* < 0.008 to adjust for multiple comparisons.

Metabolite	Mean concentration (mM) ± SD	CRLB (mM)	Age	Alcohol		
			Pearson's r	*p*-value	Partial r	*p*-value
Total NAA	11.36 ± 1.25	3.03	0.26	0.164	−0.15	0.414
Total creatine	9.23 ± 1.53	3.64	−0.34	0.059	**−0.49**	**0.006**
Total choline	2.43 ± 0.46	5.52	−0.41	0.022	−0.33	0.074
Myo-inositol	10.95 ± 2.79	4.85	−0.25	0.169	−0.36	0.053
Glutamate	7.12 ± 1.43	12.45	−0.18	0.338	−0.05	0.812
Glutamine	4.05 ± 1.12	26.79	0.04	0.826	−0.18	0.353
Tissue properties						
GM (%)	54.4 ± 10					
WM (%)	29 ± 13					
CSF (%)	16 ± 0.7					

Abbreviation: CRLB, Cramér-Rao lower bounds.

## 4 Discussion

This study aimed to examine the role of age and alcohol consumption on hippocampal metabolite levels in older adults. In our sample of 37 healthy older adults, we did not find an association between age and metabolite levels in the hippocampus. Alcohol consumption, on the other hand, was negatively correlated with creatine levels.

Despite failing to reach significance, tCr and tCho levels showed a tendency to decrease with age. Across the brain, previous reports of changes in creatine with age have been largely inconsistent. In a systematic review of 33 studies, creatine was reported to increase 14 times, decrease 6 times and to remain unchanged 13 times (Cleeland et al., 2019). Some of this inconsistency may be due to variations in voxel locations. For example, in one study, age and tCr were negatively associated in the basal ganglia, while being positively associated in the parietal voxel (Sailasuta et al., 2008). Similarly, Zahr et al. (2013) found that tCr decreased with age in the striatum, but increased in the cerebellum. It is, therefore, plausible that ageing affects creatine differently in different brain regions.

Voxel location cannot fully explain this volatile pattern of results, however. Hippocampal MRS studies have reported increased (Chiu et al., 2014) and decreased NAA (Driscoll et al., 2003; Szentkuti et al., 2004), unchanged tCr (Chiu et al., 2014) and increased mI (Reyngoudt et al., 2012) with age. The decrease of tCr is of particular relevance given its common use as a “stable denominator” for metabolite ratios. We would therefore caution against using creatine as a reference metabolite – particularly in studies using multiple VOIs and studies encompassing a wide age span in their cohort. It is also worth noting that, in contrast to several studies describing a decrease in NAA with ageing (Cleeland et al., 2019), we observed a positive correlation coefficient between NAA and age in our sample. A similar finding has recently been reported in a hippocampal MRS study (Sporn et al., 2019). While interesting, we are cautious to interpret this finding given that it did not approach significance and is in discordance with the majority of the literature to date.

Our findings suggest that cohort differences in lifestyle factors, such as alcohol consumption, could also account for inconsistencies in previous findings. Alcohol consumption was negatively correlated with tCr in the hippocampus. Decreased creatine reflects an overall decline in energy metabolism (Rae, 2014). Unlike in the parent cohort (Topiwala et al., 2017), hippocampal volume was not associated with alcohol consumption in this sub-sample (Appendix 4). This is likely a result of the reduced power in our smaller sub-sample. Taken together, this pattern of results suggests that alcohol consumption may lead to an overall decline in cell energy metabolism in a period when neuronal damage has not yet occurred. In addition, it is possible that creatine levels may be more sensitive to alcohol than hippocampal volume. Given the association between alcohol consumption and tCr levels, it may be advisable for future MRS studies to account for drinking variables in their analyses.

The key strength of our study was the use of ultra-high field imaging, which enabled us to measure concentrations of metabolites which pose technical challenges at lower field strengths (e.g. glutamate) in a technically challenging region (the hippocampus). Our study also has some limitations that must be kept in mind. Firstly, our alcohol consumption questionnaire was collected up to 3 months before the MRS data collection and we can therefore not rule out the possibility that participants may have changed their typical drinking behaviour in this period of time. However, in the Whitehall II Imaging sub-study, the larger cohort from which our sample was drawn from, drinking patterns were remarkably consistent over time, with no significant change in alcohol consumption over the 30 year study period (Topiwala et al., 2017). In addition, including time interval between questionnaire and scan as a covariate did not alter the pattern of results (Appendix 5). A further consideration is our sample size, which may have resulted in our analysis being underpowered. While certainly small, our sample size is in line with previous hippocampal MRS studies in older adults at 1.5T and 3T (N_range_ = 24–35) (Angelie et al., 2001; Chiu et al., 2014; Driscoll et al., 2003; Schuff et al., 1999; Szentkuti et al., 2004), with one exception (*N* = 90; 30 were over the age of 60) (Reyngoudt et al., 2012). It is also worth noting that, given the additional safety contraindications for 7T scanning (e.g. certain surgical implants are considered safe at 3T but as yet there is insufficient evidence on their safety at 7T), the MRI safety screening will have increased the inevitable healthy bias in our sample. Finally, since the Whitehall II cohort consists of British civil-servants recruited in the 1980s, a working-force which was then predominantly male, our sample was almost entirely male. Given that sex differences in brain metabolite levels have been demonstrated (Hjelmervik et al., 2018), suggesting our findings may not be generalizable across sexes, we repeated our analysis after excluding the women in the sample (Appendix 6). Although associations were attenuated, potentially due to the smaller sample size, the same pattern of results was observed.

In summary, driven by our previous findings of an effect of alcohol on hippocampal volume, we examined the association between alcohol consumption and MRS measures. Our findings indicated an association between tCr levels and alcohol, which in turn emphasises the importance of considering alcohol consumption as a covariate in MRS research before differences in tCr are attributed to ageing or disease. We did not find a significant association between hippocampal metabolite levels and age in this sample of healthy older adults. The ability to measure brain metabolites in vivo has the potential of furthering our understanding of the ageing brain and may even reveal candidate neurochemical processes suitable for targeting in healthy-ageing interventions. It is crucial, however, that we first identify the factors which have the potential to obfuscate any age-related effects on brain metabolite levels.

## Declaration of Competing Interest

None

## Appendix Supplementary materials

Image, application 1

Image, application 2

## References

[bib0001] Angelie E., Bonmartin A., Boudraa A., Gonnaud P.M., Mallet J.J., Sappey-Marinier D. (2001). Regional differences and metabolic changes in normal aging of the human brain: proton MR spectroscopic imaging study. AJNR Am. J. Neuroradiol..

[bib0002] Bednarik P., Moheet A., Deelchand D.K., Emir U.E., Eberly L.E., Bares M., Seaquist E.R., Oz G. (2015). Feasibility and reproducibility of neurochemical profile quantification in the human hippocampus at 3 T. NMR Biomed.

[bib0003] Buhler M., Mann K. (2011). Alcohol and the human brain: a systematic review of different neuroimaging methods. Alcohol Clin. Exp. Res..

[bib0004] Chiu P.W., Mak H.K., Yau K.K., Chan Q., Chang R.C., Chu L.W. (2014). Metabolic changes in the anterior and posterior cingulate cortices of the normal aging brain: proton magnetic resonance spectroscopy study at 3 T. Age.

[bib0006] Department of Health, 1995. Sensible drinking: the report of an inter-departmental working group, London.

[bib0005] Cleeland C., Pipingas A., Scholey A., White D. (2019). Neurochemical changes in the aging brain: a systematic review. Neurosci. Biobehav. Rev..

[bib0007] Driscoll I., Hamilton D.A., Petropoulos H., Yeo R.A., Brooks W.M., Baumgartner R.N., Sutherland R.J. (2003). The aging hippocampus: cognitive, biochemical and structural findings. Cereb. Cortex.

[bib0008] Eylers V.V., Maudsley A.A., Bronzlik P., Dellani P.R., Lanfermann H., Ding X.Q. (2016). Detection of normal aging effects on human brain metabolite concentrations and microstructure with whole-brain MR spectroscopic imaging and quantitative MR imaging. AJNR Am. J. Neuroradiol..

[bib0009] Filippini N., Zsoldos E., Haapakoski R., Sexton C.E., Mahmood A., Allan C.L., Topiwala A., Valkanova V., Brunner E.J., Shipley M.J., Auerbach E., Moeller S., Ugurbil K., Xu J.Q., Yacoub E., Andersson J., Bijsterbosch J., Clare S., Griffanti L., Hess A.T., Jenkinson M., Miller K.L., Salimi-Khorshidi G., Sotiropoulos S.N., Voets N.L., Smith S.M., Geddes J.R., Singh-Manoux A., Mackay C.E., Kivimaki M., Ebmeier K.P. (2014). Study protocol: the Whitehall II imaging sub-study. BMC Psychiatry.

[bib0011] Fotuhi M., Do D., Jack C. (2012). Modifiable factors that alter the size of the hippocampus with ageing. Nat. Rev. Neurol..

[bib0012] Gao F., Barker P.B. (2014). Various MRS application tools for Alzheimer disease and mild cognitive impairment. AJNR Am. J. Neuroradiol..

[bib0013] Hjelmervik H., Hausmann M., Craven A.R., Hirnstein M., Hugdahl K., Specht K. (2018). Sex- and sex hormone-related variations in energy-metabolic frontal brain asymmetries: a magnetic resonance spectroscopy study. Neuroimage.

[bib0014] Lee E., Jang D.P., Kim J.J., An S.K., Park S., Kim I.Y., Kim S.I., Yoon K.J., Namkoong K. (2007). Alteration of brain metabolites in young alcoholics without structural changes. Neuroreport.

[bib0015] Lemke C., Hess A., Clare S., Bachtiar V., Stagg C., Jezzard P., Emir U. (2015). Two-voxel spectroscopy with dynamic B0 shimming and flip angle adjustment at 7 T in the human motor cortex. NMR Biomed.

[bib0016] Mon A., Durazzo T.C., Meyerhoff D.J. (2012). Glutamate, GABA, and other cortical metabolite concentrations during early abstinence from alcohol and their associations with neurocognitive changes. Drug Alcohol Depend.

[bib0017] Oz G., Tkac I. (2011). Short-echo, single-shot, full-intensity proton magnetic resonance spectroscopy for neurochemical profiling at 4 T: validation in the cerebellum and brainstem. Magn. Reson. Med..

[bib0018] Provencher S.W. (2001). Automatic quantitation of localized in vivo 1H spectra with LCModel. NMR Biomed.

[bib0019] Quadrelli S., Mountford C., Ramadan S. (2016). Hitchhiker's guide to voxel segmentation for partial volume correction of in vivo magnetic resonance spectroscopy. Magn. Reson. Insights.

[bib0020] Rae C.D. (2014). A guide to the metabolic pathways and function of metabolites observed in human brain 1H magnetic resonance spectra. Neurochem. Res..

[bib0021] Revelle W. (2018).

[bib0022] Reyngoudt H., Claeys T., Vlerick L., Verleden S., Acou M., Deblaere K., De Deene Y., Audenaert K., Goethals I., Achten E. (2012). Age-related differences in metabolites in the posterior cingulate cortex and hippocampus of normal ageing brain: a 1H-MRS study. Eur. J. Radiol..

[bib0023] Team RStudio (2016). RStudio: Integrated Development for R.

[bib0024] Sailasuta N., Ernst T., Chang L. (2008). Regional variations and the effects of age and gender on glutamate concentrations in the human brain. Magn. Reson. Imaging.

[bib0025] Schuff N., Amend D.L., Knowlton R., Norman D., Fein G., Weiner M.W. (1999). Age-related metabolite changes and volume loss in the hippocampus by magnetic resonance spectroscopy and imaging. Neurobiol. Aging.

[bib0038] Sporn L., MacMillan E., Ge R., Greenway K., Villa-Rodriguez F., Laule C. (2019). Longer Repetition Time Proton MR Spectroscopy Shows Increasing Hippocampal and Parahippocampal Metabolite Concentrations with Aging. J. Neuroimaging.

[bib0026] Suri S., Emir U., Stagg C.J., Near J., Mekle R., Schubert F., Zsoldos E., Mahmood A., Singh-Manoux A., Kivimaki M., Ebmeier K.P., Mackay C.E., Filippini N. (2017). Effect of age and the APOE gene on metabolite concentrations in the posterior cingulate cortex. Neuroimage.

[bib0027] Szentkuti A., Guderian S., Schiltz K., Kaufmann J., Munte T.F., Heinze H.J., Duzel E. (2004). Quantitative MR analyses of the hippocampus: unspecific metabolic changes in aging. J. Neurol..

[bib0028] Tkac I., Andersen P., Adriany G., Merkle H., Ugurbil K., Gruetter R. (2001). In vivo 1H NMR spectroscopy of the human brain at 7 T. Magn. Reson. Med..

[bib0029] Topiwala A., Allan C.L., Valkanova V., Zsoldos E., Filippini N., Sexton C., Mahmood A., Fooks P., Singh-Manoux A., Mackay C.E., Kivimaki M., Ebmeier K.P. (2017). Moderate alcohol consumption as risk factor for adverse brain outcomes and cognitive decline: longitudinal cohort study. BMJ.

[bib0030] Tunc-Skarka N., Weber-Fahr W., Ende G. (2015). Recreational alcohol use induces changes in the concentrations of choline-containing compounds and total creatine in the brain: a (1)H MRS study of healthy subjects. MAGMA.

[bib0031] van de Bank B.L., Emir U.E., Boer V.O., van Asten J.J., Maas M.C., Wijnen J.P., Kan H.E., Oz G., Klomp D.W., Scheenen T.W. (2015). Multi-center reproducibility of neurochemical profiles in the human brain at 7 T. NMR Biomed.

[bib0032] Voets N.L., Hodgetts C.J., Sen A., Adcock J.E., Emir U. (2017). Hippocampal MRS and subfield volumetry at 7T detects dysfunction not specific to seizure focus. Sci. Rep..

[bib0033] Wickham H., Sievert C. (2016). ggplot2 : Elegant Graphics for Data Analysis.

[bib0034] Wilson S., Bair J.L., Thomas K.M., Iacono W.G. (2017). Problematic alcohol use and reduced hippocampal volume: a meta-analytic review. Psychol Med.

[bib0035] Zahr N.M., Mayer D., Rohlfing T., Chanraud S., Gu M., Sullivan E.V., Pfefferbaum A. (2013). In vivo glutamate measured with magnetic resonance spectroscopy: behavioral correlates in aging. Neurobiol. Aging.

[bib0036] Zahr N.M., Mayer D., Rohlfing T., Hasak M.P., Hsu O., Vinco S., Orduna J., Luong R., Sullivan E.V., Pfefferbaum A. (2010). Brain injury and recovery following binge ethanol: evidence from in vivo magnetic resonance spectroscopy. Biol. Psychiatry.

[bib0037] Zhang Y., Brady M., Smith S. (2001). Segmentation of brain MR images through a hidden Markov random field model and the expectation-maximization algorithm. IEEE Trans. Med. Imaging.

