# Supplementary material for: Alcohol consumption is associated with reduced creatine levels in the hippocampus of older adults
Source: Psychiatry Res. 2020 Jan 30;295:111019. doi: 10.1016/j.pscychresns.2019.111019 (PMC6961205; doi:10.1016/j.pscychresns.2019.111019)
Supplement: Supplementary file 2 [file mmc2.docx]

**Supplementary material**

**Appendix 1**. Correlations between hippocampal metabolite concentrations, as ratios to tCr, and age (univariate) and weekly alcohol consumption (bivariate; covariate = age).

| **Metabolite** | **Age** | | **Alcohol** | |
| --- | --- | --- | --- | --- |
|  | Pearson’s r | *p*-value | Partial r | *p*-value |
| tNAA/tCr | **0.5** | **0.005** | **0.54** | **0.002** |
| tCho/tCr | -0.16 | 0.385 | 0.22 | 0.246 |
| mI/tCr | -0.13 | 0.495 | -0.13 | 0.486 |
| Glu/tCr | 0.1 | 0.587 | 0.46 | 0.011 |
| Gln/tCr | 0.27 | 0.149 | 0.17 | 0.383 |

**Appendix 2.** Sample demographics of the Whitehall II 3T MRI cohort and the 7T MRS sub-sample. To assess if the 7T sub-sample was representative of the larger cohort, we ran two-tailed t-tests for continuous variables and *X*^2^ for categorical variables (number of women).

|  | 3T MRI sample | 7T MRS sub-sample | t (or *X*^2^) | p |
| --- | --- | --- | --- | --- |
| *Demographics* |  |  |  |  |
| *n* | 800 | 31 |  |  |
| Age (years) | 69.9 ± 5.2 | 70.4 ± 5.6 | -0.56 | 0.58 |
| Sex (N, % Female) | 152, 23.5% | 3, 9.6% | 1.15 | 0.28 |
| Education (years) | 14.7 ± 2.4 | 16 ± 2.9 | **-2.38** | **0.02** |
| MoCA | 27.2 ± 2.3 | 27.4 ± 2.1 | -0.68 | 0.5 |

Values are mean ± standard deviation.

**Appendix 3.** Plots to illustrate the zero-order associations between [tCr] and weekly alcohol consumption (*r* = -0.47, *p* = 0.009) and [tCr] and age (*r* = -0.34, *p* = 0.059). In addition, zero-order correlations between metabolites and age are presented.


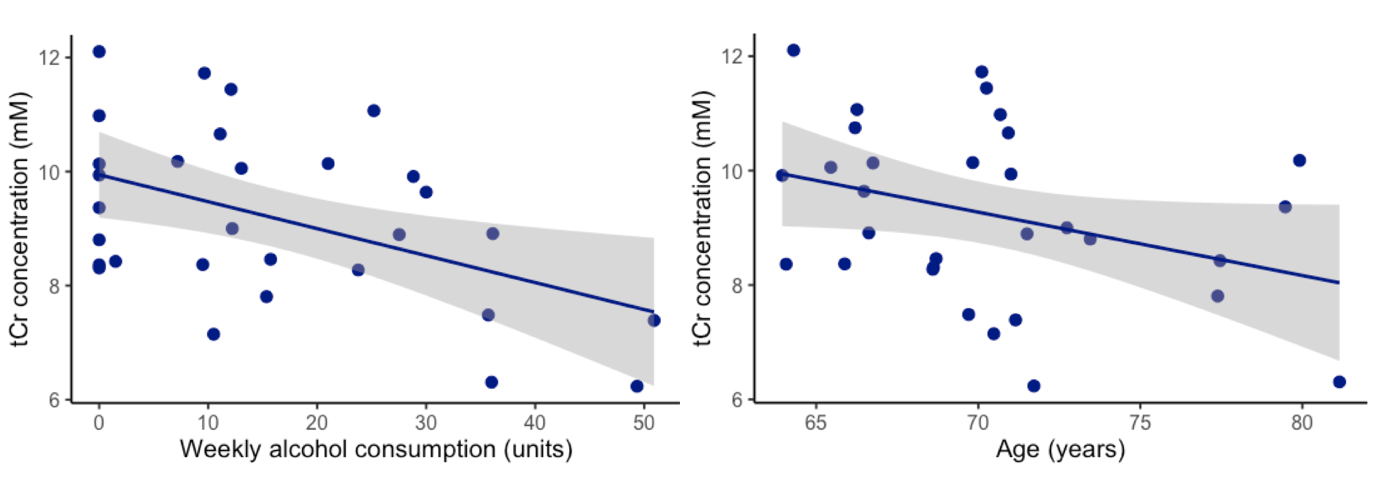


| **Metabolite** | **Alcohol** | |
| --- | --- | --- |
|  | r | *p*-value |
| tNAA | -0.14 | 0.449 |
| tCr | -0.47 | 0.009 |
| tCho | -0.31 | 0.094 |
| mI | -0.35 | 0.057 |
| Glu | -0.05 | 0.798 |
| Gln | -0.17 | 0.356 |

**Appendix 4.** There was no association between hippocampal volume and alcohol consumption.

Methods and results

To quantify hippocampal volume, automated segmentation of the hippocampus was performed on the T1 images using FreeSurfer v6.0 (Fischl et al., 2002). In order to adjust for total intracranial volume (ICV), hippocampal volume was expressed as a percent of ICV. Mean right hippocampal volume was 3085 mm^3^ (SD 381.56). After adjusting for age, right hippocampal volume (% ICV) was not associated with alcohol consumption (r_partial_ = -0.08, p = 0.676).

**Appendix 5.** Given that our sample was predominantly male (90.4%), and that sex differences in brain metabolite levels have been demonstrated, we repeated our analyses after excluding the data from the three female participants.

Metabolite concentrations and tissue content within the voxel are expressed as mean ± standard deviations. For each metabolite, correlations with age (univariate) and weekly alcohol consumption (bivariate; covariate = age) are shown. Significance level was set at p < 0.008 to adjust for multiple comparisons.

| **Metabolite** | **Age** | | **Alcohol** | |
| --- | --- | --- | --- | --- |
|  | Partial r | *p*-value | Partial r | *p*-value |
| tNAA | 0.39 | 0.041 | -0.05 | 0.804 |
| tCr | -0.27 | 0.161 | -0.48 | 0.01 |
| tCho | -0.37 | 0.056 | -0.36 | 0.061 |
| mI | -0.17 | 0.383 | -0.42 | 0.027 |
| Glu | -0.13 | 0.521 | -0.07 | 0.739 |
| Gln | 0.15 | 0.444 | -0.12 | 0.554 |

**Appendix 6.** Partial correlations between hippocampal metabolite concentrations and age (covariate = time interval) and weekly alcohol consumption (covariate = age, time interval). Time interval represents the lag, in days, between the measurement of alcohol consumption and the 7T MRS scan.

| **Metabolite** | **Age** | | **Alcohol** | |
| --- | --- | --- | --- | --- |
|  | Partial r | *p*-value | Partial r | *p*-value |
| tNAA | 0.25 | 0.192 | -0.15 | 0.445 |
| tCr | -0.39 | 0.034 | -0.47 | 0.010 |
| tCho | -0.41 | 0.023 | -0.33 | 0.084 |
| mI | -0.32 | 0.08 | -0.32 | 0.092 |
| Glu | -0.17 | 0.369 | -0.05 | 0.791 |
| Gln | 0 | 0.979 | -0.15 | 0.451 |

**References**

Fischl, B., Salat, D. H., Busa, E., Albert, M., Dieterich, M., Haselgrove, C., . . . Dale, A. M. (2002). Whole brain segmentation: automated labeling of neuroanatomical structures in the human brain. *Neuron, 33*(3), 341-355. Retrieved from <http://www.ncbi.nlm.nih.gov/pubmed/11832223>
